# Effects of simultaneous multislice acceleration on the stability of radiomics features in parametric maps of IVIM and DKI in uterine cervical cancer

**DOI:** 10.1002/acm2.70063

**Published:** 2025-03-02

**Authors:** Shuangquan Ai, Wei Peng, Rong Hou, Huiting Zhang, Robert Grimm, Zilong Yuan, Yulin Liu

**Affiliations:** ^1^ Department of Radiology Hubei Cancer Hospital, Tongji Medical College, Huazhong University of Science and Technology Wuhan China; ^2^ College of Biomedical Engineering South‐Central Minzu University Wuhan Hubei China; ^3^ Department of Patholoogy Suizhou Hospital Affiliated to Hubei Medical College Shiyan Hubei China; ^4^ MR Scientific Marketing, Siemens Healthineers Wuhan China; ^5^ MR Application Predevelopment, Siemens Healthcare GmbH Erlangen Germany

**Keywords:** cervical cancer, diffusion kurtosis imaging, intravoxel incoherent motion, radiomics features, stability

## Abstract

**Purpose:**

The aim of this study was to investigate the influence of the simultaneous multislice acceleration (SMS) technique as well as two‐dimensional (2D) and three‐dimensional (3D) tumor segmentations on radiomics features (RFs) within the parametric maps of cervical cancer, which were computed by intravoxel incoherent motion (IVIM) and diffusion kurtosis imaging (DKI). Additionally, the study sought to identify those RFs that could characterize the clinical stages (low‐stage vs. high‐stage) of cervical cancer.

**Materials and methods:**

Multi‐*b*‐value diffusion‐weighted imaging (DWI) of 40 patients with cervical cancer were collected using the SMS technique with acceleration factors (AF) of 1–3. RFs were extracted from parametric maps representing pure diffusion coefficient (D), pseudodiffusion coefficient (D*), perfusion fraction (f), mean diffusivity (MD), and mean kurtosis (MK). A total of 93 2D and 93 3D RFs were extracted from per parametric map. The concordance correlation coefficient (CCC) and coefficients of variation (COV) were used to jointly assess the stability of features. Finally, the intra‐class correlation coefficient (ICC) was used for intra‐group consistency assessment. Receiver operating characteristic (ROC) curve was used to evaluate diagnostic performance of stable features in distinguishing lower and higher stages.

**Results:**

Feature stability decreased with higher AF. Among these features, 9.1% of 2D and 12.7% of 3D RFs were stable (CCC > 0.9 and COV ≤ 0.1). ADC maps had the highest stability, whileas D^*^ and f maps had the lowest stability and 3D features were more stable than 2D features. A total of 5 2D and 25 3D stable features were identified that could distinguish lower and higher stages (AUC = 0.66–0.83).

**Conclusion:**

SMS demonstrated impact on the stability of RFs in IVIM and DKI parametric maps, particularly for D* and f maps. Multi‐*b*‐value DWI with SMS (AF = 2) was recommended for clinical radiomics research.

## 1 INTRODUCTION

Cervical cancer is one of the most common malignancies of the female reproductive system, ranking fourth worldwide in both incidence and mortality among cancers affecting women.^1^ The clinical stage of cervical cancer is an indication of tumor invasiveness and has a considerable impact on patient treatment planning and prognostic evaluation.^2^ The International Federation of Obstetrics and Gynecology (FIGO) divides cervical cancer into four stages (stage I‐ IV), with stage II further subdivided into IIA and IIB. These stages indeed have a significant impact on the treatment approach for cervical cancer. In patients with stage I‐IIA, surgical treatment is usually used to preserve ovarian and vaginal functions of patients, while, for patients with stage IIB‐IV, concurrent chemoradiotherapy is mainly used for comprehensive treatment.^2^ The clinical staging of cervical cancer typically depends on cervical histopathologic biopsy. However, the histopathology‐based staging standard is invasive, discomfort‐causing, and recurrence‐risky. Moreover, it requires a long waiting time for obtaining pathologic results.^3^ Thus, a noninvasive and highly effective method is urgently required in the clinical setting.

Presently, both intravoxel incoherent movement (IVIM) and diffusion kurtosis imaging (DKI) have been used for the preoperative staging and efficacy assessment of cervical cancer.^4^, ^5^, ^6^ Radiomics, as an emerging field, enriches these techniques by extracting quantitative features from imaging data to capture the heterogeneity of tumors. Several studies have explored combining radiomics with parametric maps for cervical cancer analysis.^7^, ^8^, ^9^, ^10^ For example, Wang et al.^8^ reported that the combination of radiomics features (RFs) extracted from T2‐weighted imaging and DKI with a random forest modeling algorithm could be used to predict the clinical stage of cervical cancer. Zhang et al.^7^ demonstrated that RFs based on IVIM parametric maps (*D* ^*^ and *f*) could be used to predict the efficacy of simultaneous radiotherapy for cervical cancer. However, the successful translation of radiomics studies to clinical applications depends heavily on the stability of the radiomic features.^11^

Both IVIM and DKI involve multi‐b‐value fitting, resulting in long acquisition times that increase the likelihood of motion artifacts and signal loss. The simultaneous multislice (SMS) acceleration technique addresses this limitation by significantly reducing acquisition time.^12^, ^13^ Simultaneous excitation and acquisition of multiple slices, facilitated by the use of an acceleration factor (AF), reduces the required scanning time by enabling the excitation of a larger number of slices simultaneously. Linear combinations of signals from each of the slices, weighted by the spatial sensitivity profiles of the coils, are then received by a radiofrequency coil channel, and matrix inversion is used to reconstruct the signal for each individual slice. In this technique, the number of slices acquired over the same pulse repetition time can be increased by setting a higher AF without the need for an increase in the gradient demand.^12^ Nevertheless, the impact of SMS on RFs derived from IVIM and DKI maps remains unexplored. Therefore, the purpose of this study was to (1) investigate stability of RFs with SMS acceleration based on IVIM and DKI model; (2) test such variations in 2D and 3D features with SMS; and (3) identify the RFs characterizing the clinical stages (low‐stage vs. high‐stage) of cervical cancer.

## 2 MATERIALS AND METHODS

### 2.1 Patient characteristics

This prospective study was approved by the ethics committee of Hubei Cancer Hospital [(2021)IEC(A033)], and the informed consent of the patient was obtained for all patients. A total of 45 patients were enrolled between April 2020 and August 2022. The inclusion criteria were as follows: (1) pathologically confirmed cervical cancer and FIGO stage in IB‐IV, without tumor history and other complications; and (2) all patients undergoing multi‐*b*‐value DWI before treatment. The exclusion criteria were as follows: (1) previous treatment prior to MRI; (2) lesion invisible on DWI images; (3) obvious susceptibility artifact affecting lesion observation; (4) visible patient movement between multiple b‐value imaging; (5) poor model fitting (*R* ^2^ < 0.8), where *R* ^2^ evaluated the goodness‐of‐fit for DKI and IVIM models. Ultimately, 40 patients with pathologically confirmed cervical cancer were prospectively included in this study (Table 1). Of these patients, 4 were diagnosed with adenocarcinoma, 36 were diagnosed with carcinoma squamous. FIGO stages were further dichotomized between lower FIGO stages (IB‐IIA) and higher FIGO stages (IIB‐IV), including 7 patients in stage IB, 5 in stage IIA, 12 in stage IIB, 14 in stage III, and 2 in stage IV.

**TABLE 1:** Demographic and tumor characteristics.

Characteristics	
Patients (*n*)	40
Age (years) (mean ± SD)	58 ± 11
Histological subtypes [*n* (%)]	
Adenocarcinoma	4 (10)
Carcinoma squamous	36 (90)
FIGO stage [*n* (%)]	
IB	7 (17.5)
IIA	5 (12.5)
IIB	12 (30.0)
IIIA	1 (2.5)
IIIB	4 (10.0)
IIIC	9 (22.5)
IVB	2 (5.0)

### 2.2 Image acquisition

In this study, all patients underwent routine pelvic examination and multi‐b‐value DWI on a 3T MRI scanner (MAGNETOM Skyra; Siemens Healthcare, Erlangen, Germany) using a 16‐channel abdomen phased array coil before treatment. In order to reduce gastrointestinal motion, anisodamine (654‐2) was administered before imaging. The scanning range from the iliac crest to the perineal. The routine examinations included sagittal T2‐weighted imaging (T2WI), axial T1‐weighted imaging (T1WI), axial high‐resolution T2WI, axial T2WI with fat suppression, and contrast‐enhanced axial T1WI, and contrast‐enhanced sagittal T1WI. Multi‐b‐value DWI used a single‐shot echo plane sequence before contrast‐enhanced axial T1WI. As depicted in Table 2, the specific parameters were as follows: multi‐b‐values range from 0 to 2000 s/mm^2^ with three orthogonal directions; field of view (FOV) = 380 × 356 mm^2^; slice thickness/space = 4/0.4 mm; and matrix = 128 × 128. Each patient was required to collect three multi‐b‐value DWI sequences (S1, S2, and S3) corresponding to the SMS AF of 1, 2, and 3, respectively. The corresponding TR/TE was 4700/82, 2500/84, and 2000/85 ms, and the acquisition time was 5 min 19 s, 2 min 58 s, and 2 min 12 s.

**TABLE 2:** Imaging parameters for multi‐b DWI sequences with different SMS acceleration factor.

Scan parameters	S1	S2	S3
Repetition time/TE (ms)	4700/82	2500/84	2000/85
Slice thickness/Space (mm)	4/0.4	4/0.4	4/0.4
Number of slice	24	24	24
Flip angle	90	90	90
Field of view (mm^2^)	380 × 356	380 × 356	380 × 356
SMS	1	2	3
Grappa	2	2	2
Fat saturation	SPAIR	SPAIR	SPAIR
Diffusion mode	3‐scan trace	3‐scan trace	3‐scan trace
Concatenations	1	1	1
*b*‐values, s/mm^2^(average)	0(1),10(1),20(1), 40(1),80(1),150(1), 200(2),400(2), 800(2),1500(4), 2000(6)	0(1),10(1),20(1), 40(1),80(1),150(1), 200(2),400(2), 800(2),1500(4), 2000(6)	0(1),10(1),20(1), 40(1),80(1),150(1), 200(2),400(2), 800(2),1500(4), 2000(6)
Bandwidth (Hz/px)	1562	1562	1562
Scanning time	5 min 19 s	2 min 58 s	2 min 12 s

### 2.3 Image quality evaluation

Considering the fact that higher b‐value provides superior tumor contrast,^14^ DWI with a b value of 800 s/mm^2^ was used for image quality evaluation. In this study, the signal‐to‐noise ratio (SNR) and contrast‐to‐noise ratio (CNR) of the lesion were measured on these images across S1, S2, and S3 sequences. The images were processed by utilizing the Siemens Syngo (VB20A) workstation. Subsequently, a radiologist who boasted 15 years of experience conducted the measurements one after another.

On the layer with largest extent of the tumor, the region of interest (ROI) for the tumor itself, the gluteus maximus muscle, and the image background were manually outlined precisely three times respectively, ensuring that the ROI maintained a consistent shape and size during the entire process. Subsequently, the signal intensity (SI) and standard deviation (SD) of the aforementioned elements, the lesion, the gluteus maximus muscle, and the background, were carefully recorded. Then, the average values were derived from these three sets of measurements. Finally, the SNR and CNR were computed based on the following formulas:

(1) $$\mathrm{SNR}=SI_{\mathrm{tumor}}\;/SD_{\mathrm{tumor}}$$

(2) $$\mathrm{CNR}=\left(SI_{\mathrm{tumor}}-SI_{\mathrm{muscle}}\right)/\sqrt{S{D_{\mathrm{tumor}}}^{2}+S{D_{\mathrm{muscle}}}^{2}}$$

where SI_tumor_ and SD_tumor_ represent the signal intensity and standard deviation on the layer with largest extent of the tumor, respectively, and SI_muscle_ and SD_muscle_ represent the signal intensity and standard deviation of the gluteus maximus muscle on the largest layer of the lesion, respectively.

### 2.4 IVIM and DKI calculation

Both IVIM and DKI parametric maps were calculated with a proprietary research application (MR Body Diffusion Toolbox v1.6.0; Siemens Healthcare, Erlangen, Germany). IVIM maps were fitted using 9 *b* values (0–800 s/mm^2^),^15^ and the fitting formula was as follows:

(3) $$\frac{s_{b}}{s_{0}}=\left(1-f\right)\times e^{-b\times D}+f\times e^{-b\times \left(D+D*\right)}$$

where *S*_0_ and *S_b_* represent the signal intensity corresponding to *b* = 0 and specific *b* values, respectively; *f* is the perfusion fraction; *D* is diffusion coefficient; and D* is pseudodiffusion coefficient.

DKI was fitted using 6 *b* values (0, 200, 400, 800, 1500, and 2000 s/mm^2^),^16^ and the apparent diffusion coefficient (ADC) was calculated using 0 and 800 s/mm^2^. The following formula was used for DKI:

(4) $$\frac{s_{b}}{s_{0}}=e^{\left(-b\times D+\frac{b^{2}\times MD^{2}\times MK}{6}\right)}$$

where *S*_0_ and *S_b_* represent the signal intensity corresponding to *b* = 0 and specific *b* values, MD is mean diffusivity, and MK is mean kurtosis.

ADC was calculated using 0 and 800 s/mm^2^, and the formula was as follows:

(5) $$\frac{s_{b}}{s_{0}}=e^{-\left(b\times ADC\right)}$$

where *S*_0_ and *S_b_* represent the signal intensity corresponding to *b* = 0 and specific *b* values.

### 2.5 ROI segmentation

**FIGURE 1:**
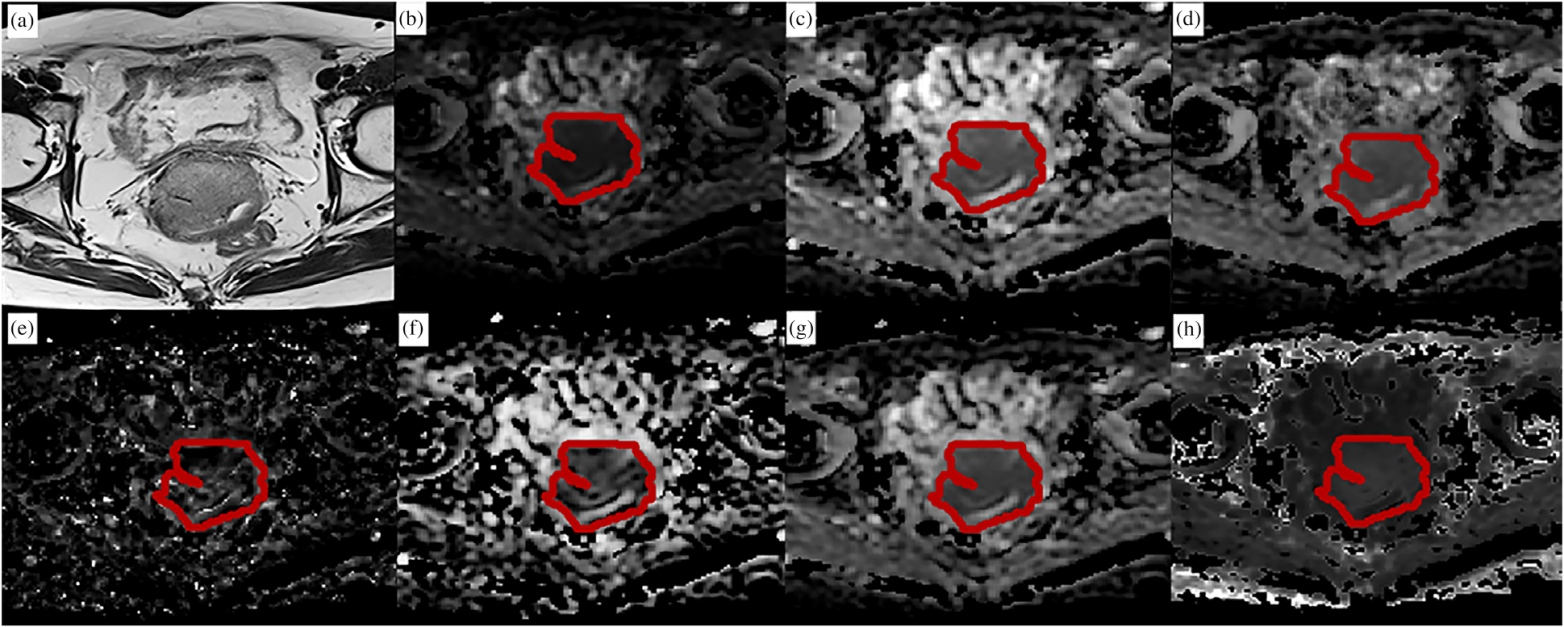
A 57‐year‐old patient with International Federation of Gynecology and Obstetrics (FIGO) stage IIB cervical cancer magnetic resonance imaging (MRI), marked with a region of interest (ROI). (a) T2WI, (b) tumor was manually drawn at the diffusion‐weighted imaging (DWI) image at *b* = 800 s/mm^2^, (c∼h) are copied contour to corresponding ADC map, D map, D* map, f map, MD map, and MK map, respectively. The average values (ADC∼MK) of the region of interest (ROI) are 900.10 × 10^−3^ mm^2^/s, 900.49 × 10^−3^ mm^2^/s, 1225.57 × 10^−3^ mm^2^/s, 93.5%, 1216.58 × 10^−3^ mm^2^/s and 854.99, respectively.

Due to the reasons mentioned above, DWI with a b value of 800 s/mm^2^ was also used for tumor segmentation, as shown in Figure 1. Referencing to the corresponding high‐resolution T2WI and contrast‐enhanced axial T1WI images, ROIs with layers with maximum axial size of the lesion (2D) and full layers (3D) of the tumor were outlined by two radiologists with 10 and 15 years of experience, respectively. ROIs with meticulous care taken to avoid the regions of hemorrhage, edema, and necrosis. Delineation was performed using open‐source Slicer software (http://www.slicer.org). The drawn ROIs were then registered to all the diffusion parametric maps of all sequences (S1–S3) for feature calculation.

### 2.6 Feature extraction

In this study, 93 RFs of each parametric map were extracted through the Pyradiomics package of Python (version 3.7.1), including the first order (*n* = 18), gray‐level co‐occurrence matrix (GLCM, *n* = 24), gray‐level run‐length matrix (GLRLM, *n* = 16), gray‐level size zone matrix (GLSZM, *n* = 16), neighborhood gray‐tone difference matrix (NGTDM, *n* = 5), and gray‐level dependence matrix (GLDM, *n* = 14). Finally, 558 2D and 558 3D RFs were obtained from the six parameters.

### 2.7 Stability evaluation

The stability of RFs was assessed using the concordance correlation coefficient (CCC) and coefficient of variation (COV). These metrics evaluate the reproducibility of features across different SMS acceleration factors (AFs).

The formula used to calculate COV was as follows:

(6) $$\mathrm{COV}\;=\frac{\sigma }{\mu }\;$$

where σ represents the standard deviation (SD) of the characteristic value, and *μ* represents the average of the combination of various parametric map features. COV was divided into four categories: poor group (COV > 0.2), moderate group (0.1 ≤ COV ≤ 0.2), good group (0.05 ≤ COV ≤ 0.1), and excellent group (COV < 0.05)^17^

The formula used to calculate CCC was as follows:

(7) $$\mathrm{CCC}=\frac{2\rho \sigma_{x}\sigma_{y}}{\sigma_{x}^{2}+\sigma_{y}^{2}+{\left(u_{x}-u_{y}\right)}^{2}}$$

where *x* and *y* are the vector values of the RFs extracted from the parametric map under the 2 AF values; and *ρ*, *σ*, and *μ* are the correlation coefficient, SD, and average of *x* and *y* vector values, respectively. CCC was also divided into four categories: poor group (CCC ≤ 0.5), moderate group (0.5 ≤ CCC ≤ 0.75), good group (0.75 ≤ CCC ≤ 0.9), and excellent group (CCC > 0.9).^18^ Origin software (version 9.9) was used to analyze the change trend of features.

The CCC of the S1‐S2 and S1‐S3 groups was computed, and the COV of the S1, S2, and S3 groups was determined to assess the stability of the RFs across parametric maps within different sequence groups. Then, the average values of CCC and COV of features in those groups were used to evaluate the impact of ROIs and AF values on the radiomics analysis. The COV and CCC characterized the changes in RFs with AF. In this study, features with COV ≤ 0.1^17^ and CCC > 0.9^18^ represented stable features.

### 2.8 Statistical analysis

The independent‐sample *t* test for data with normal distribution and Mann–Whitney *U* test for data with non‐normal‐distribution, were used to identify RFs that could characterize the clinical stage (low‐stage vs. high‐stage) of cervical cancer. Objective image quality evaluation was conducted using the Friedmen test, and for groups with significant differences, pairwise comparisons were carried out using the Dunn‐Bonferroni post hoc test. The intra‐class correlation coefficient (ICC) was also used to assess the impact of inter‐observer effects for these selected features, ICC > 0.75 represent excellent interobserver characteristic agreement. Subsequently, the diagnostic performance of these features in distinguishing lower between higher stages was assessed using receiver operating characteristic (ROC) curve. A *p* value < 0.05 indicated a statistically significant difference.

## 3 RESULTS

### 3.1 Image quality assessment

Table 3 summarizes image quality assessment of DWI with a b‐value of 800 s/mm^2^ between different SMS. For the SNR and CNR, there was no statistically significant difference between S1 and S2 (SNR: 144.27 ± 35.70 vs. 148.41 ± 43.95, *p* = 0.937; CNR: 16.05 ± 0.90 vs. 15.59 ± 1.29, *p* = 1.000). S1 was higher than S3 with a statistically significant difference (SNR: 144.27 ± 35.70 vs. 126.30 ± 35.38, *p* = 0.035; CNR: 16.05 ± 0.90 vs. 11.78 ± 0.64, *p* < 0.001). S2 was also higher than S3 with a statistically significant difference (SNR: 148.41 ± 43.95 vs. 126.30 ± 35.38, *p* = 0.001; CNR: 15.59 ± 1.29 vs. 11.78 ± 0.64, *p* < 0.001).

**TABLE 3:** Image quality assessment of DWI with a *b*‐value of 800 s/mm^2^ between different SMS.

	S1	S2	S3	*p*	P1	P2	P3
SNR	144.27 ± 35.70	148.41 ± 43.95	126.30 ± 35.38	<0.001	0.937	0.001	0.035
CNR	16.05 ± 0.90	15.59 ± 1.29	11.78 ± 0.64	<0.001	1.000	<0.001	<0.001

*Note*: P1: the post hoc comparison between S1 and S2; P2: the post hoc comparison between S2 and S3; P3: the post hoc comparison between S1 and S3.

Abbreviations: CNR, contrast to noise ratio; SNR, signal noise ratio.

### 3.2 Stability of changes in RFs with the AF

**FIGURE 2:**
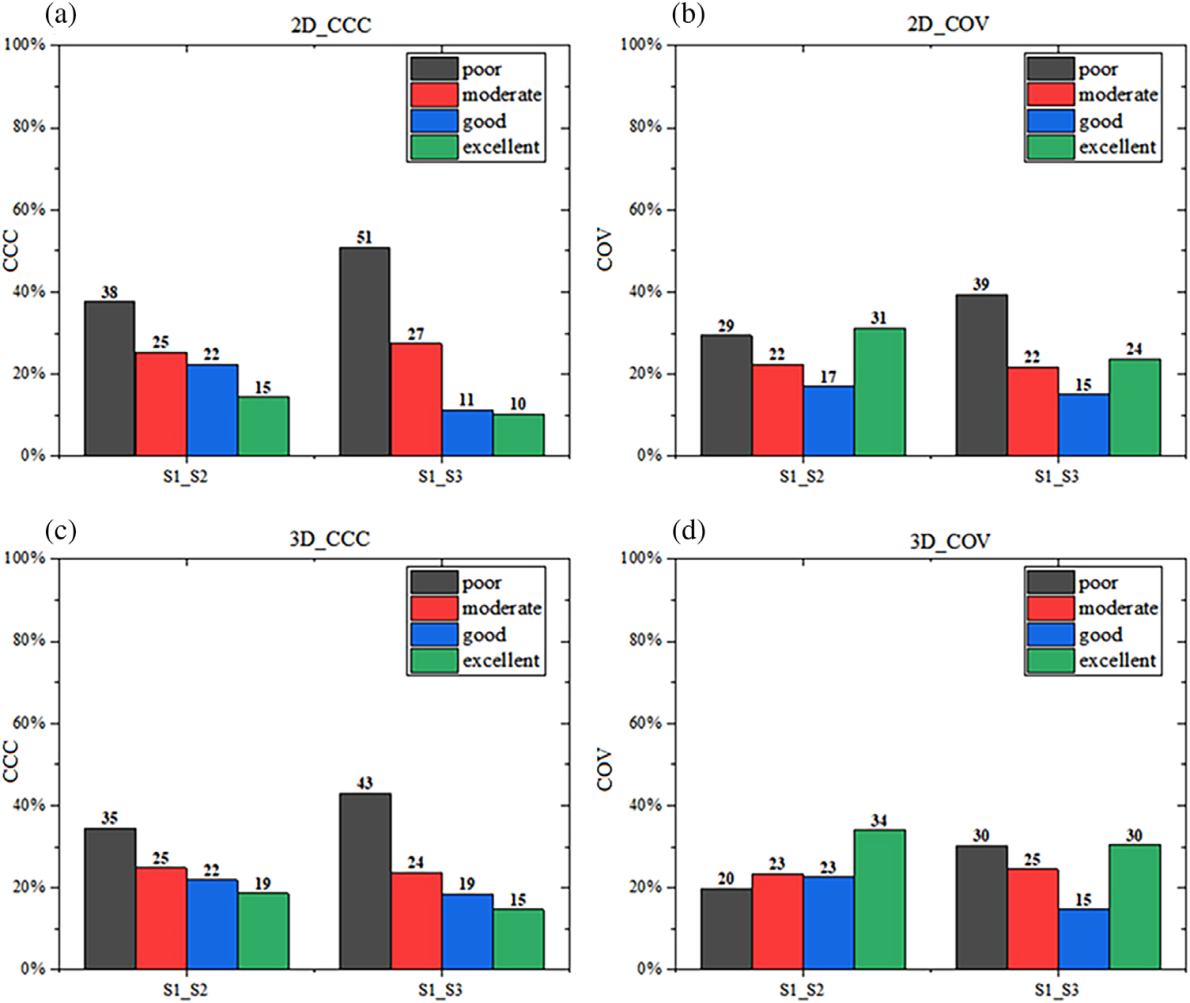
Proportions of various categories of concordance correlation coefficient (CCC) and coefficient of variation (COV) across all parametric maps, based on 2D (*n* = 558) and 3D (*n* = 558) radiomics features derived from sequences S1‐S2 and S1‐S3. The area of each column represents the proportion of the total number of features. The four groups were defined as follows: poor group (CCC ≤ 0.5 and COV > 0.2), moderate group (0.5 ≤ CCC ≤ 0.75 and 0.1 ≤ COV ≤ 0.2), good group (0.75 ≤ CCC ≤ 0.9 and 0.05 ≤ COV ≤ 0.1), and excellent group (CCC > 0.9 and COV < 0.05).

Figure 2 illustrates the proportion of various CCC and COV categories across all maps. For 2D features extracted from the 6 sets of parametric maps (*n* = 558), the proportion of the excellent category in CCC and COV decreased from 15% and 31% to 10% and 24%, and that of the poor category increased from 38% and 29% to 51% and 39%, respectively, with the increase in AF (Panels a and b). The trend for 3D features (*n* = 558) was similar to that for 2D features. The proportion of the excellent category decreased from 19% and 34% to 15% and 30% and that of the poor category increased from 35% and 20% to 43% and 30%, respectively (Panels c and d).

**FIGURE 3:**
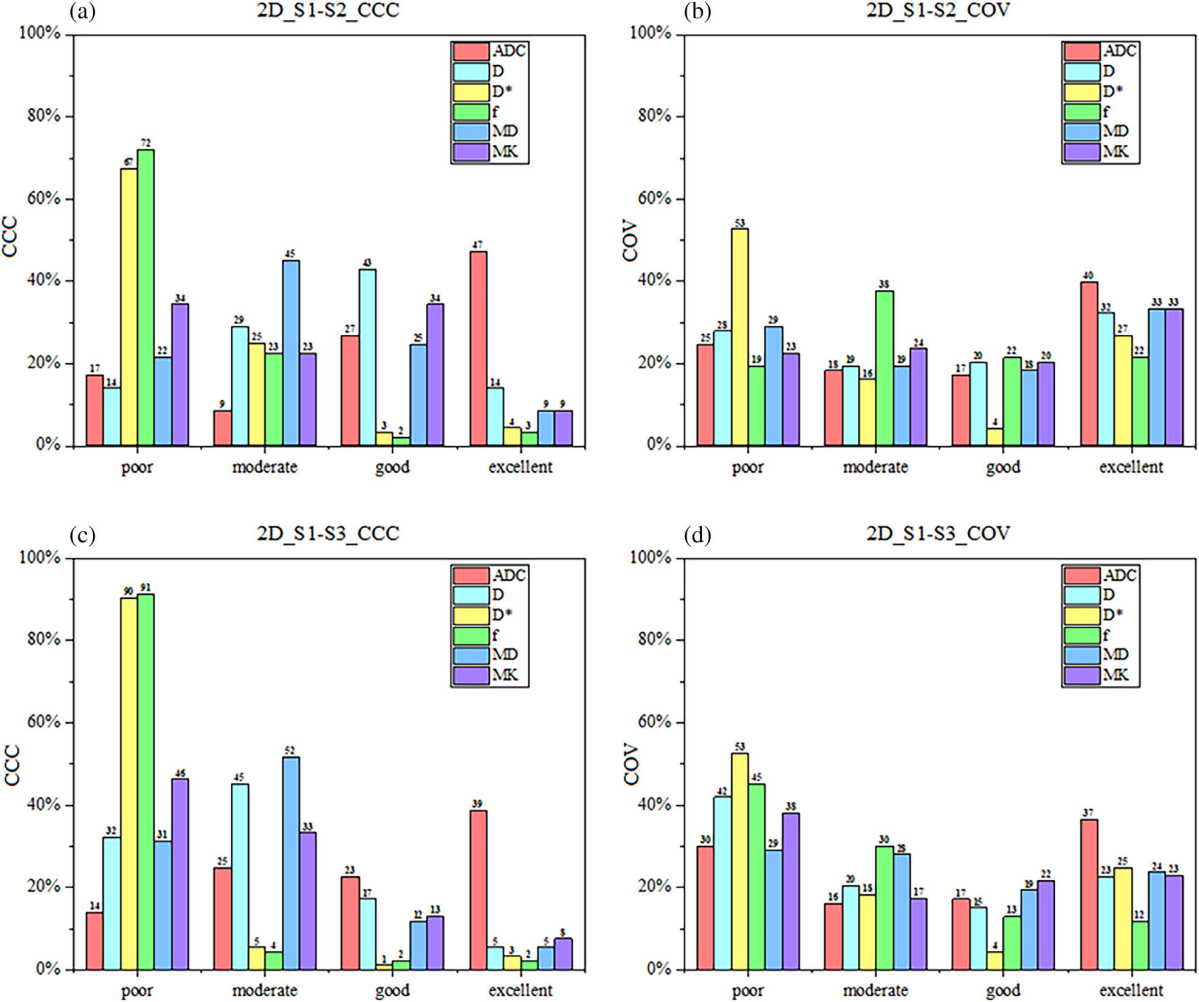
Proportions of various categories of concordance correlation coefficient (CCC) and coefficient of variation (COV) across various parametric maps, based on 2D radiomics features (*n* = 93) derived from sequences S1‐S2 and S1‐S3. The area of each column represents the proportion of the total number of features.

Figure 3 depicts the proportion of various CCC and COV categories in parametric maps according to 2D RFs (*n* = 93). Panels a and b demonstrate the CCC comparison results between S1 and S2 sequences. The ADC map had the highest proportion of the excellent category in both CCC (47%) and COV (40%) analyses. The *D* ^*^ and *f* maps revealed the highest proportion of the poor category, accounting for 67% and 72% in CCC and 53% and 19% in COV, respectively. Panels c and d also demonstrate the comparison results between S1 and S3 sequences. Similar results were also observed for the ADC map, but with a lower proportion of the excellent category for CCC (39%) and COV (37%) analyses, compared with the results obtained from the comparison between S1 and S2 sequences. The D* and *f* maps also demonstrated a higher proportion of poor category, with 90% and 91% for CCC and 53% and 45% for COV.

**FIGURE 4:**
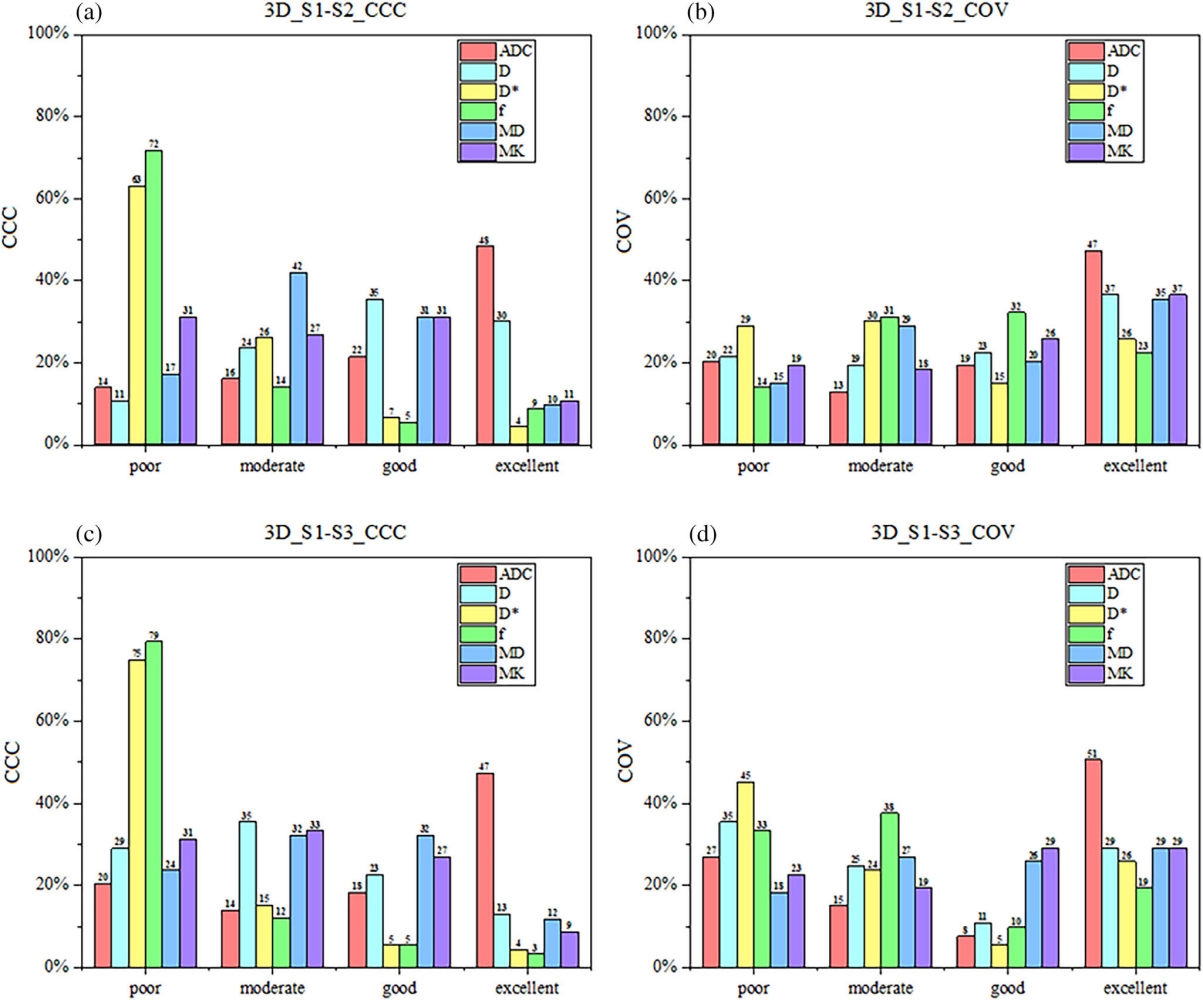
Proportions of various categories of concordance correlation coefficient (CCC) and coefficient of variation (COV) across various parametric maps, based on 3D radiomics features (*n* = 93) derived from sequences S1‐S2 and S1‐S3. The area of each column represents the proportion of the total number of features.

The 3D RFs are also depicted in Figure 4. As depicted in Panels a and b, of 93 RFs in each parametric map, ADC map demonstrated a higher proportion of excellent category in CCC (48%) and COV (47%) analyses between S1 and S2 sequences. The *D* ^*^ and *f* maps revealed the highest proportion of the poor category, accounting for 67% and 72% in CCC and 29% and 14% in COV, respectively. Panels c and d depict the comparison results between S1 and S3 sequences. The same trend was observed in the ADC map, with the highest proportion of excellent category (CCC, 47%; COV, 51%). The *D* ^*^ and *f* maps also revealed a lower proportion of the poor category, accounting for 75% and 79% in CCC and 45% and 33% in COV, compared with 2D RFs.

### 3.3 Evaluation of stable features

**FIGURE 5:**
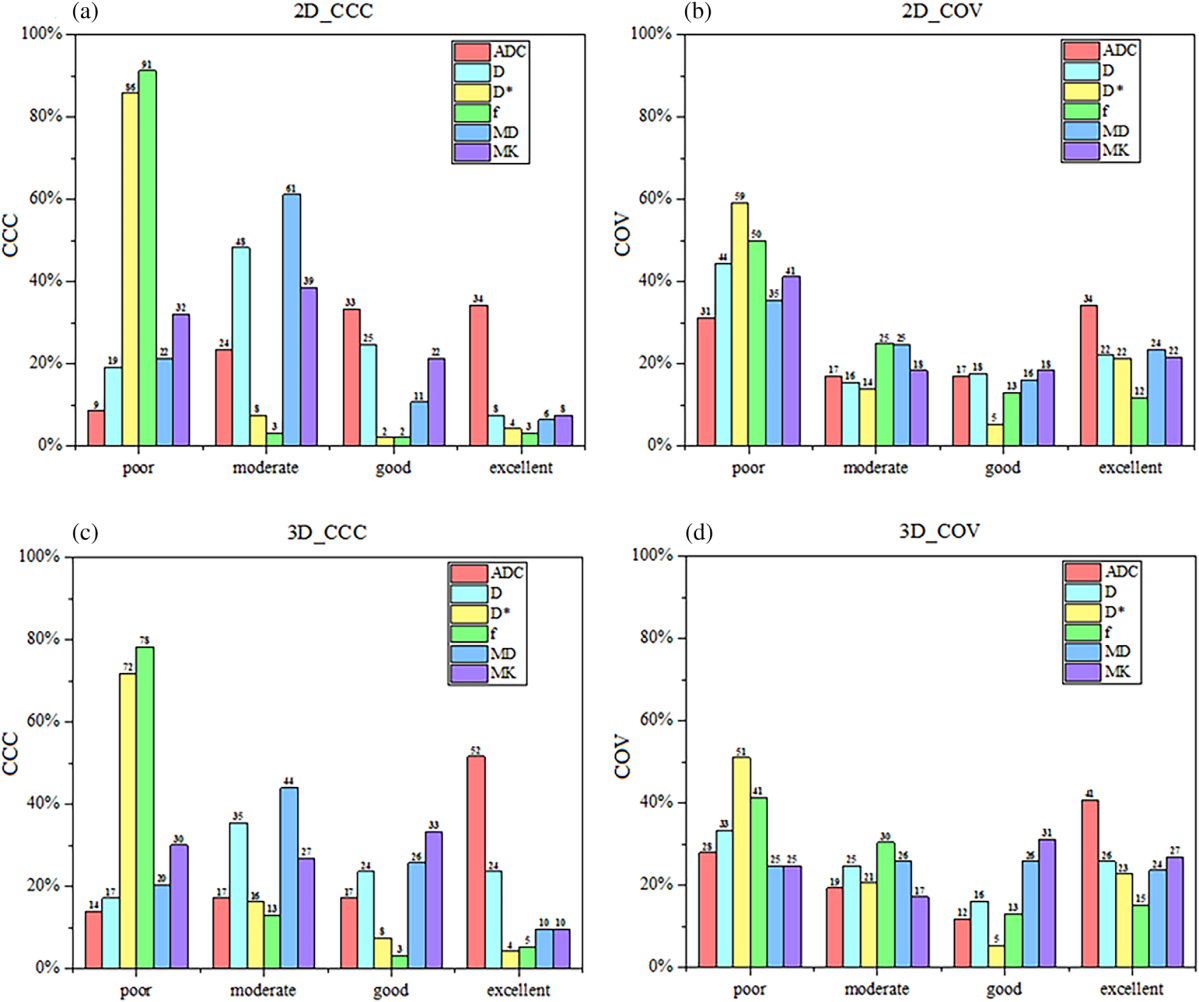
Proportion of different categories of concordance correlation coefficient (CCC) and coefficient of variation (COV) under various parametric maps across 2D versus 3D radiomics features (*n* = 93). a and b represent 2D radiomics features. c and d represent 3D radiomics features.

Figure 5 depicts the proportion of CCC and COV categories in parametric maps across 2D versus 3D RFs (*n* = 93 for each map). The proportions of 2D RFs are depicted in Panels a and b; the proportion of excellent category in CCC and COV for the ADC, *D*, *D* ^*^, *f*, MD, and MK was 34% and 34%, 8% and 22%, 4% and 22%, 3% and 12%, 6% and 24%, and 8% and 22%, respectively. Panels c and d present the proportion of 3D RFs. The proportion of excellent category in CCC and COV for the ADC, *D*, *D* ^*^, *f*, MD, and MK was 57% and 41%, 8% and 22%, 4% and 22%, 3% and 12%, 6% and 24%, and 8% and 22%, respectively.

**FIGURE 6:**
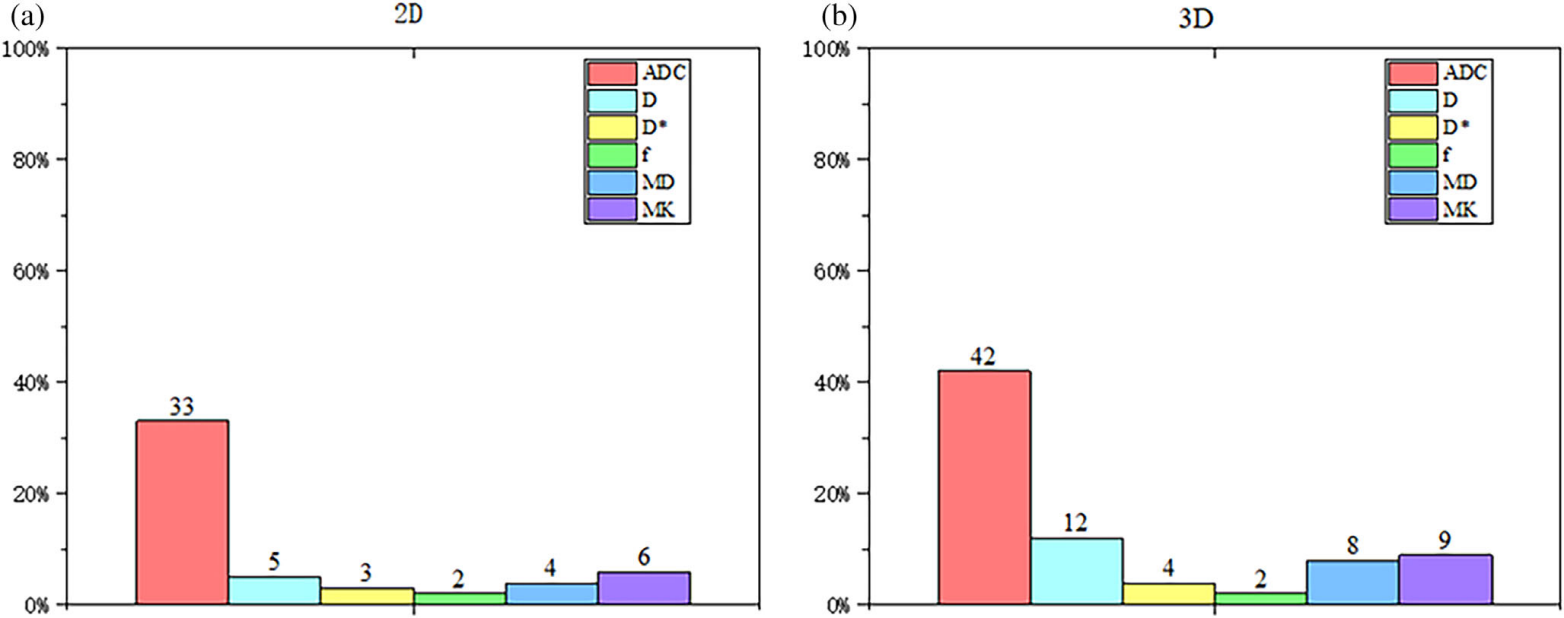
Proportion of stable features (CCC > 0.9 and COV ≤ 0.1) in 2D versus 3D radiomics features (*n* = 93) for various maps. A represent the stability features of 2D radiomics features, and B represent the stability features of 3D radiomics features. The area of each column reflects the proportion of the total number of features.

**FIGURE 7:**
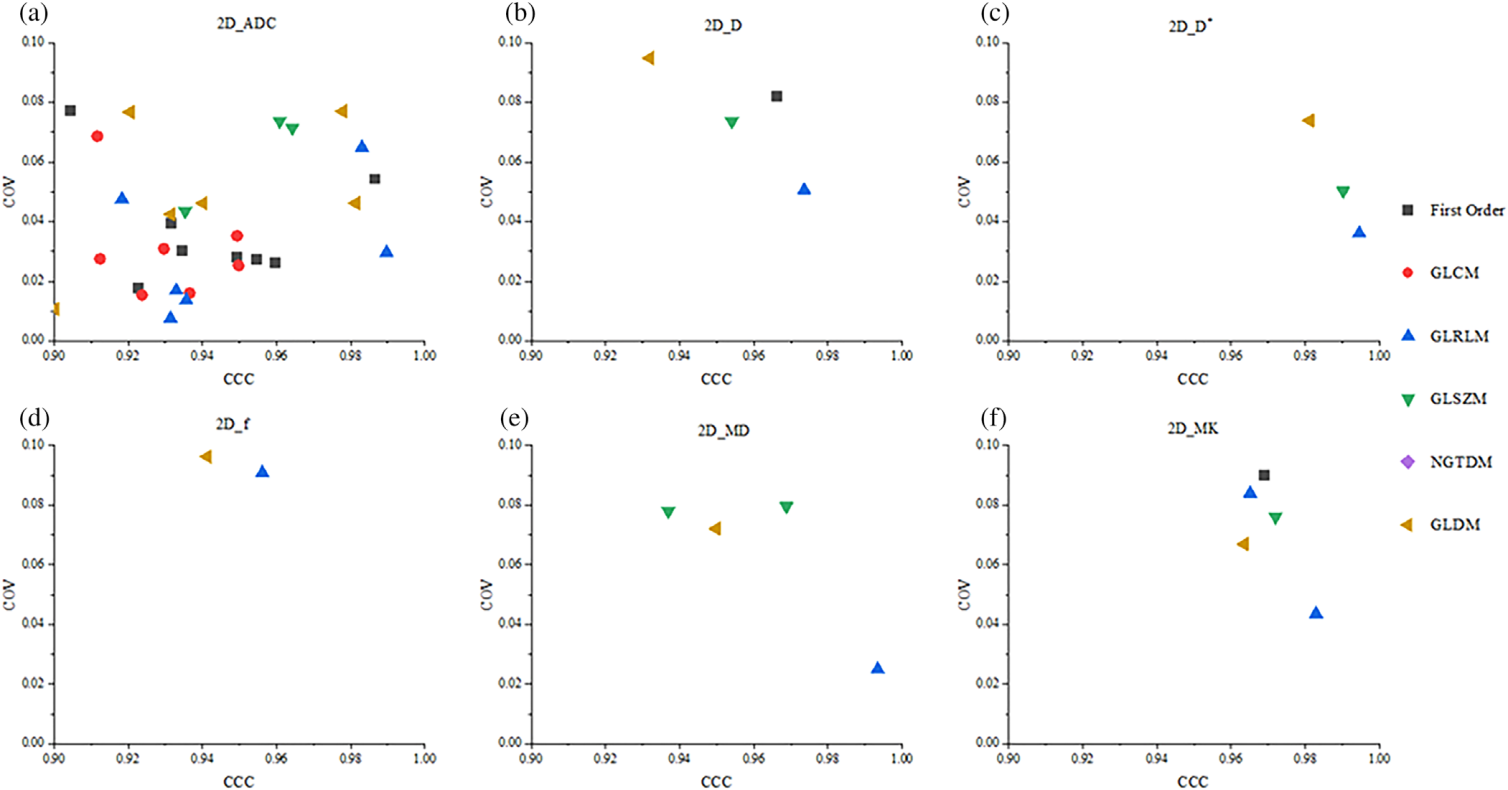
Scatter plot of 2D stable radiomics features that could distinguish between six categories of disease stages across multiple maps.

**FIGURE 8:**
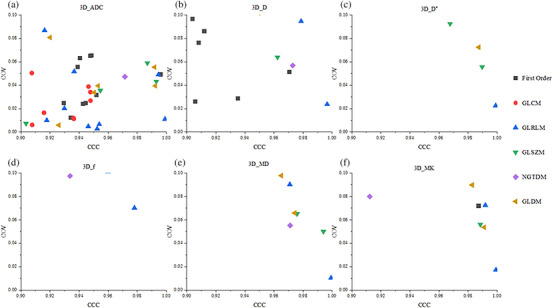
Scatter plot of 3D stable radiomics features that could distinguish between six categories of disease stages across multiple maps.

The proportion of stable features (CCC > 0.9 and COV ≤ 0.1) is depicted in Figure 6. For 2D RFs (*n* = 93 for each map), the ADC map demonstrated the highest proportion of stable features (33%); the other maps presented a relatively low proportion (2%–6%). Although 3D features (*n* = 93 for each map) provided a higher proportion than 2D features, the ADC maps exhibited 42% of stable features. Also, 12%, 4%, 2%, 8%, and 9% stable features were observed for *D*, *D* ^*^, *f*, MD, and MK, respectively. We also generated scatter plots to display the distribution of CCC and COV for each feature in six categories of features to better illustrate the distribution of feature categories in stable features. As depicted in Figures 7 and 8, the ADC map present the most stable features in both 2D (31) and 3D (39) RFs. Compared with other categories of RFs, the first order (2D and 3D; 13 and 20), GLCM (2D and 3D; 9 and 8), and GLRLM (2D and 3D; 12 and 18) categories accounted for more stable features.

### 3.4 Features related to FIGO stage

By comparing the image quality and total scanning time needed of S1, S2, and S3, S2 was used for subsequent analysis. Based on the aforementioned analysis, there are 51 2D features and 71 3D features which are stable in these quantitative maps. Of these features, only 26 2D features and 46 3D features could be used to distinguish the lower stages from the higher stages (*p* < 0.05). The ICC was then used to assess the impact of inter‐observer effects. Finally, 5 2D features and 26 3D features exhibited excellent interobserver agreement (ICC > 0.75). The AUC values for these selected 2D features range from 0.66 to 0.76, and 3D features range from 0.70 to 0.83. Table 4 lists these selected RFs that could characterize the clinical stage of cervical cancer. Specifically, of those 2D features, two were exacted from the ADC map, one from the f map, and two from the MK map. In 3D features, six from the ADC map, three from the D map, four from the D^*^, one from the f map, four from the MD map, eight from the MK map.

**TABLE 4:** The selected stability features in lower stage and higher stage in different maps, as well as the *p*‐value and AUC value with FIGO staging.

	Map	Category	Feature	Lower stage	Higher stage	*p*	AUC
2D	ADC	FirstOrder	10Percentile	6.768 × 10^2^ ± 1.437 × 10^2^	7.695 × 10^2^ ± 1.266 × 10^2^	0.048	0.702
			Median	8.322 × 10^2^ ± 1.640 × 10^2^	9.578 × 10^2^ ± 1.707 × 10^2^	0.037	0.669
	f	GLRLM	GrayLevelNonUniformity	58.246 (46.413,1.106 × 10^2^)	1.086 × 10^2^ (72.325,1.635 × 10^2^)	0.034	0.705
	MK	GLRLM	GrayLevelNonUniformity	31.463(24.754,53.858)	60.587(43.082,1.227 × 10^2^)	0.010	0.764
		GLSZM	GrayLevelNonUniformity	19.789(14.940,34.542)	34.097(23.161,46.458)	0.030	0.720
3D	ADC	FirstOrder	Mean	8.915 × 10^2^ ± 1.524 × 10^2^	1.053 × 10^3^ ± 1.560 × 10^2^	0.004	0.752
			Median	8.407 × 10^2^ ± 1.553 × 10^2^	9.634 × 10^2^ ± 1.559 × 10^2^	0.013	0.731
			RootMeanSquared	8.959 × 10^2^ (8.215 × 10^2^,9.495 × 10^2^)	1.068 × 10^3^ (9.883 × 10^2^,1.168 × 10^3^)	0.001	0.755
		GLRLM	GrayLevelNonUniformity	2.408 × 10^2^ (1.204 × 10^2^,4.770 × 10^2^)	4.922 × 10^3^ (2.819 × 10^2^,1.050 × 10^3^)	0.027	0.717
		GLSZM	GrayLevelNonUniformity	86.864 (57.654,1.296 × 10^2^)	1.588 × 10^2^ (1.031 × 10^2^,2.989 × 10^2^)	0.023	0.738
		GLDM	GrayLevelNonUniformity	2.718 × 10^2^ (1.286 × 10^2^,5.675 × 10^2^)	5.571 × 10^2^ (3.154 × 10^2^,1.213 × 10^3^)	0.034	0.705
	D	GLRLM	GrayLevelNonUniformity	2.041 × 10^2^ (93.248,3.891 × 10^2^)	4.404 × 10^2^ (2.433 × 10^2^,9.213 × 10^2^)	0.030	0.708
			RunLengthNonUniformity	1.150 × 10^4^ (7.489 × 10^3^,1.572 × 10^4^)	2.111 × 10^4^ (1.662 × 10^3^,5.659 × 10^4^)	0.004	0.800
		GLSZM	GrayLevelNonUniformity	91.098 (58.876,1.477 × 10^2^)	1.746 × 10^2^ (1.092 × 10^2^,4.468 × 10^2^)	0.032	0.711
	D*	GLRLM	RunLengthNonUniformity	1.125 × 10^4^ (7.471 × 10^3^,1.670 × 10^4^)	2.317 × 10^4^ (1.707 × 10^4^,6.239 × 10^4^)	0.003	0.785
		GLSZM	GrayLevelNonUniformity	55.122 (34.825,85.649)	1.078 × 10^2^ (75.108,2.651 × 10^2^)	0.012	0.770
			SizeZoneNonUniformity	7.967 × 10^3^ (5.204 × 10^3^,1.122 × 10^4^)	1.601 × 10^4^ (1.027 × 10^4^,4.090 × 10^4^)	0.008	0.770
		GLDM	DependenceNonUniformity	7.051 × 10^3^ (4.018 × 10^3^,9.626 × 10^3^)	1.359 × 10^4^ (9.046 × 10^3^,3.395 × 10^4^)	0.009	0.764
	f	GLRLM	RunLengthNonUniformity	8.244 × 10^3^ (5.956 × 10^3^,1.284 × 10^4^)	1.619 × 10^4^ (1.248 × 10^4^,4.577 × 10^4^)	0.003	0.770
	MD	GLRLM	GrayLevelNonUniformity	1.179 × 10^2^ (57.736,2.256 × 10^2^)	2.466 × 10^2^ (1.377 × 10^2^,5.158 × 10^2^)	0.023	0.744
			RunLengthNonUniformity	1.128 × 10^4^ (7.773 × 10^3^,1.669 × 10^4^)	2.267 × 10^4^ (1.175 × 10^4^,6.143 × 10^4^)	0.003	0.791
		GLSZM	GrayLevelNonUniformity	71.996 (39.772,1.172 × 10^2^)	1.461 × 10^2^ (89.932,3.452 × 10^2^)	0.022	0.750
		GLDM	GrayLevelNonUniformity	1.230 × 10^4^ (61.459,2.387 × 10^2^)	2.587 × 10^2^ (1.523 × 10^2^,5.340 × 10^2^)	0.020	0.750
	MK	FirstOrder	Energy	1.131 × 10^10^ (6.650 × 10^9^,2.026 × 10^10^)	1.986 × 10^10^ (1.414 × 10^10^,5.134 × 10^10^)	0.043	0.702
			TotalEnergy	1.061 × 10^10^ (6.234 × 10^9^,1.899 × 10^10^)	1.862 × 10^10^ (1.326 × 10^10^,4.813 × 10^10^)	0.043	0.702
		GLRLM	GrayLevelNonUniformity	2.469 × 10^2^ (1.336 × 10^2^,3.616 × 10^2^)	6.353 × 10^2^ (3.441 × 10^2^,1.452 × 10^3^)	0.003	0.824
			RunLengthNonUniformity	1.082 × 10^4^(7.245 × 10^3^,1.554 × 10^4^)	1.928 × 10^4^(1.489 × 10^4^,5.363 × 10^4^)	0.008	0.758
		GLSZM	GrayLevelNonUniformity	92.629 (60.794,1.638 × 10^2^)	1.811 × 10^2^(1.215 × 10^2^,3.758 × 10^2^)	0.017	0.752
		GLDM	DependenceNonUniformity	2.789 × 10^3^(2.227 × 10^3^,4.437 × 10^3^)	4.141 × 10^3^(3.529 × 10^3^,1.048 × 10^4^)	0.030	0.708
			GrayLevelNonUniformity	2.938 × 10^2^(1.446 × 10^2^,3.871 × 10^2^)	7.078 × 10^2^(4.129 × 10^2^,1.700 × 10^3^)	0.001	0.836

*Note*: Normal distribution data are presented as mean ± SD, non‐normally distributed data are presented as median (25–75th percentiles). *p* < 0.05 represents a statistical difference.

## 4 DISCUSSION

This study investigated the impact of the SMS technique and different manual segmentation approaches on RFs in the parametric maps based on IVIM and DKI models of cervical cancer. It also aimed to select features from stable features that could characterize the FIGO staging of cervical cancer. The results of this study demonstrated that the proportion of stable features decreased with increasing AF of SMS. The SMS technique had the greatest effect on *D* ^*^ and *f* maps, whereas ADC maps were relatively stable. Meanwhile, 3D RFs demonstrated higher stability than 2D features. The RFs extracted from the parametric maps of SMS (AF = 2) based on IVIM and DKI models could effectively characterize the FIGO staging of cervical cancer.

Conventional diffusion and multi‐*b*‐value diffusion have been widely used in radiomics studies.^19^, ^20^, ^21^ Further, few studies also investigated the stability of the features of these images in depth. For example, Gitto et al.^22^ assessed the feature stability through small geometric transformations of the ROIs mimicking multiple manual delineations. They discovered that 76.4% (1300/1702) of RFs were stable on the ADC map for spinal bone tumors. Granzier et al.^23^ evaluated the stability of the RFs of T2WI, T1WI, and ADC map of breast volunteers with varying image preprocessing (bias field correction, z‐score normalization, and grayscale correction) using test–retest data. They demonstrated that images without preprocessing produced the highest proportion of stable features for T1W sequence and ADC maps, with 16.5% (15/91) and 8.8% (8/91) of stable features, respectively. Meanwhile, Traverso et al.^24^ demonstrated that normalization together with a smaller bin before feature extraction could improve the stability of ADC RFs from 63% (348/552) to 78% (428/552). A small percentage of studies also investigated the effects of imaging parameters on the stability of RFs. For example, He et al.^17^ explored the impact of variable *b*‐value combinations on ADC‐based RFs. The results indicated that only 20% (18/92) of RFs were robust against the variations in *b*‐value combinations with COV < 5%. Dreher et al.^25^ investigated the stability of RFs in EPI‐DWI at various resolutions (2 × 2  and 3 × 3 mm^2^) using intra‐/interobserver reproducibility and test–retest robustness evaluation. They demonstrated that DWI provided robust RFs, with those obtained from ADC being slightly less robust than those obtained from raw DWI (*b* = 500 and 1000 s/mm^2^). Furthermore, no substantial difference was detected across various resolutions. However, studies on the effect of SMS on the stability of RFs extracted from IVIM and DKI maps have not yet been reported.

In this study, SMS greatly decreased 44%–59% of the acquired time for IVIM and DKI imaging, which rendered multi‐*b*‐value diffusion imaging more suitable for clinical examination. However, this reduction in imaging time might unavoidably entail a certain degree of image quality loss. Park et al.^12^ demonstrated that the signal‐to‐noise ratio decreased considerably with the increase in AF in SMS, potentially affecting signal accuracies. Xu et al.^15^ also demonstrated that lower TR times due to higher AF might lead to signal losses because of T1 saturation effects. Our study observed the greatest impact on the RFs from *D* ^*^ and *f* maps than other maps. The possible reason was that *D* ^*^ represented the slope of the curve of the low‐*b*‐value fitting part in IVIM. The slight change in signal intensity on the low‐*b*‐value diagram caused by SMS might lead to an abrupt change in the slope and consequently cause a notable change in the *D* ^*^ diagram. This, in turn, can considerably affect the RFs. *f* represents the fraction of the change in dispersion coefficient caused by microcirculation in the total dispersion coefficient, which is highly related to *D* ^*^. Therefore, SMS also greatly impacted the features of the *f* diagram. Other parameters such as ADC, D, MD, and MK were fitted with relatively high *b* values, and SMS had a relatively small impact on high‐b‐value images and fitting results. Thus, it had relatively less influence on the features. Of course, although SMS had a certain impact on RFs, considering the total scanning time needed and the fact that there are still many features that were stable at AF = 2, we still recommend using SMS (AF = 2) for relevant radiomics research. In addition, in the ADC diagram, we revealed that the first‐order features based on 3D segmentation accounted for the most stable features (11/18, 61%), and the NGTDM accounted for the least number (1/5, 20%) (Figure 8). One possible reason was that the first‐order features were often histogram‐based, discriminating gray‐level signal intensities within an ROI without considering spatial relationships between neighboring voxels. On the contrary, the texture features such as GLDM, GLCM, GLRLM, NGTDM, and so forth, identified the spatial relationship between gray‐level signal intensities by constructing a gray‐level dependence matrix. As a result, the texture features exhibited a higher sensitivity to varying sequences and external imaging modalities. Gourtsoyianni et al.^26^ demonstrated that the stability of first‐order features was higher than that of texture features on T2WI images, and GLRLM, GLSZM, and NGTDM categories had the lowest stability, which was consistent with our findings.

Several studies demonstrated that the 2D RFs were more sensitive than the 3D features. This led to lower *z*‐axis resolution and easily produced artifacts compared with those on the *x*‐ and *y*‐axes.^27^ Zhao et al.^28^ demonstrated that the 3D features were more responsive to tumor heterogeneity than the 2D features. Gitto et al.^27^ demonstrated that the 3D RFs were more stable than the 2D features in Ewing's sarcoma. Meanwhile, Schurink et al.^29^ confirmed that the stability of RFs was higher in 3D segmentation than in 2D segmentation in multicenter rectal cancer data, whether segmented by experts or nonexperts. In addition, Fasmer et al.^30^ demonstrated that 3D RFs could better predict the FIGO staging of endometrial cancer compared with 2D RFs. All these results demonstrated that the 3D features are more stable than the 2D features, which was consistent with our results. Meanwhile, we detected more 3D features that could be used to distinguish the FIGO stage of cervical cancer compared with 2D RFs, which also proved that the 3D RFs could better represent the heterogeneity within the tumor.

A few studies have reported that IVIM‐ or DKI‐based radiomics can be used to distinguish subtypes and grades,^31^ and predict stage,^8^ treatment response,^7^ recurrence, and disease‐free survival (DFS)^10^ for cervical cancer. For example, Zhang et al.^31^ evaluated the subtypes and grades of cervical cancer based on the DKI‐based texture feature model. They demonstrated that the combined map of kurtosis along the axial direction (Ka), kurtosis along the axial direction (Kr), and MD performed the best in differentiating cervical adenocarcinoma from squamous carcinoma [area under the curve (AUC) = 0.932]. Zhang et al.^7^ predicted the sensitivity of concurrent chemotherapy (CCRT) for locally advanced cervical cancer using IVIM‐DWI combined with clinical prognostic impact factors. The results indicated that all parametric maps except *D*‐value images had diagnostic values with AUCs of 0.987 and 0.984 in the training and test groups, respectively. Zhang et al.^10^ predicted survival after simultaneous radiotherapy for advanced cervical cancer based on IVIM parametric map using nomogram combined with clinical and imaging RFs. *D* and *f* were highly associated with DFS in cervical cancer. Zhao et al.^32^ used MRI‐based radiomics to establish imaging‐based classifiers so as to distinguish the early stages of cervical cancer and demonstrated that six features from the ADC, including three textural features (GLDM, *n* = 1; GLSZM, *n* = 2) and three first‐order features (kurtosis, *n* = 1; skewness, *n* = 2) could be used to identify early stages. Wang et al.^33^ assessed the histologic subtype, tumor grade, FIGO stage, and lymph node status of cervical cancer using texture features on T2WI, ADC, and contrast‐enhanced T1WI. The corresponding AUC was 0.841, 0.850, 0.898, and 0.879, respectively. The results also indicated that the mean and entropy extracted from ADC maps were highly correlated with FIGO staging, and these results were partially compatible with our findings (Table 4), which could be used in practice to improve cervical cancer diagnosis and treatment.

This study had certain limitations. First, our sample was relatively small. However, we obtained a few valuable and stable features that could be used to predict the clinical staging of cervical cancer. Second, although we excluded cases with visibly noticeable motion between various SMS sequences, a slight motion that cannot be detected visually may still exist between these sequences, leading to an inaccurate evaluation of feature stability. Additionally, SMS itself might introduce potential errors (e.g., signal saturations due to crosstalk in the adjacent slices, residual aliasing/slice leakage, chemical shift, and so on),^34^, ^35^, ^36^, ^37^, ^38^ all of which may affect the accuracy of the results. In addition, we did not investigate the influence of automatic segmentation and manual segmentation. This aspect has currently emerged as a research hotspot, and we plan to conduct further research in the future. Furthermore, this study only evaluated the stability of the most commonly used clinical FirstOrder and texture features. However, a large number of imaging studies use higher‐order features or deep learning features to model imaging histology. Therefore, an in‐depth analysis of such features is needed to ensure the stability of the model. Finally, only cervical cancer was evaluated in this study, future research underwent SMS should be focused on other types of cancer or exploring the use of other advanced imaging techniques in combination with radiomics analysis.

In conclusion, this study demonstrated that both SMS and the tumor delineation method had effects on RFs in cervical cancer based on IVIM and DKI models, particularly in 2D segmentation and maps related to *D* ^*^ and *f*. In addition, multi‐*b*‐value DWI based on SMS (AF = 2) can be recommended for clinical radiomics research, RFs extracted from these parametric maps based on IVIM and DKI models could effectively characterize the FIGO staging (lower and higher) of cervical cancer.

## AUTHOR CONTRIBUTIONS

**Shuangquan Ai**: Conceptualization, data curation, formal analysis investigation, methodology, software, visualization, writing‐original draft writing‐review & editing. **Wei Peng**, **Rong Hou**, **Huiting Zhang**. **Robert Grimm**: Methodology, supervision; **Yulin Liu** and **Zilong Yuan** (Corresponding Author): Conceptualization, supervision, validation, writing‐original draft, writing‐review & editing.

## CONFLICT OF INTEREST STATEMENT

The authors declare that they have no competing interests.

## ETHICS STATEMENT

This prospective study was approved by the ethics committee of Hubei Cancer Hospital [(2021)IEC(A033)], and the informed consent of the patient was obtained for all patients.

## Data Availability

All relevant data and materials have been included in the article. Further inquiries can be directed to the corresponding authors.

## References

[1] Bray F , Ferlay J , Soerjomataram I , Siegel RL , Torre LA , Jemal A . Global cancer statistics 2018: gLOBOCAN estimates of incidence and mortality worldwide for 36 cancers in 185 countries. CA Cancer J Clin. 2018;68(6):394‐424. 30207593 10.3322/caac.21492

[2] Kaur H , Silverman PM , Iyer RB , Verschraegen CF , Eifel PJ , Charnsangavej C . Diagnosis, staging, and surveillance of cervical carcinoma. AJR Am J Roentgenol. 2003;180(6):1621‐1631. 12760933 10.2214/ajr.180.6.1801621

[3] Salvo G , Odetto D , Pareja R , Frumovitz M , Ramirez PT . Revised 2018 international federation of gynecology and obstetrics (FIGO) cervical cancer staging: A review of gaps and questions that remain. Int J Gynecol Cancer. 2020;30(6):873‐878. 32241876 10.1136/ijgc-2020-001257

[4] Zhang Q , Ouyang H , Ye F , et al. Feasibility of intravoxel incoherent motion diffusion‐weighted imaging in distinguishing adenocarcinoma originated from uterine corpus or cervix. Abdom Radiol. 2021;46(2):732‐744. 10.1007/s00261-020-02586-4 32671441

[5] Song J , Lu Y , Wang X , et al. A comparative study of four diffusion‐weighted imaging models in the diagnosis of cervical cancer. Acta Radiol. 2022;63(4):536‐544. 33745294 10.1177/02841851211002017

[6] Wang P , Thapa D , Wu G , Sun Q , Cai H , Tuo F . A study on diffusion and kurtosis features of cervical cancer based on non‐Gaussian diffusion weighted model. Magn Reson Imaging. 2018;47:60‐66. 29103978 10.1016/j.mri.2017.10.016

[7] Zhang Y , Zhang K , Jia H , et al. IVIM‐DWI and MRI‐based radiomics in cervical cancer: Prediction of concurrent chemoradiotherapy sensitivity in combination with clinical prognostic factors. Magn Reson Imaging. 2022;91:37‐44. 35568271 10.1016/j.mri.2022.05.005

[8] Wang M , Perucho JAU , Vardhanabhuti V , Ip P , Ngan HYS , Lee EYP . Radiomic features of T2‐weighted imaging and diffusion kurtosis imaging in differentiating clinicopathological characteristics of cervical carcinoma. Acad Radiol. 2022;29(8):1133‐1140. 34583867 10.1016/j.acra.2021.08.018

[9] Wang M , Perucho JAU , Chan Q , et al. Diffusion kurtosis imaging in the assessment of cervical carcinoma. Acad Radiol. 2020;27(5):e94‐e101. 31324577 10.1016/j.acra.2019.06.022

[10] Zhang Y , Liu L , Zhang K , et al. Nomograms combining clinical and imaging parameters to predict recurrence and disease‐free survival after concurrent chemoradiotherapy in patients with locally advanced cervical cancer. Acad Radiol. 2023;30(3):499‐508. 36050264 10.1016/j.acra.2022.08.002

[11] Ford J , Dogan N , Young L , Yang F . Quantitative radiomics: impact of pulse sequence parameter selection on MRI‐based textural features of the brain. Contrast Media Mol Imaging. 2018;2018:1729071. 30154684 10.1155/2018/1729071 PMC6091359

[12] Park JH , Seo N , Lim JS , Hahm J , Kim MJ . Feasibility of simultaneous multislice acceleration technique in diffusion‐weighted magnetic resonance imaging of the rectum. Korean J Radiol. 2020;21(1):77‐87. 31920031 10.3348/kjr.2019.0406 PMC6960306

[13] Filli L , Ghafoor S , Kenkel D , et al. Simultaneous multi‐slice readout‐segmented echo planar imaging for accelerated diffusion‐weighted imaging of the breast. Eur J Radiol. 2016;85(1):274‐278. 26547123 10.1016/j.ejrad.2015.10.009

[14] Padhani AR , Liu G , Mu‐Koh D , et al. Diffusion‐weighted magnetic resonance imaging as a cancer biomarker: Consensus and recommendations. Neoplasia. 2009;11:102‐125. 19186405 10.1593/neo.81328 PMC2631136

[15] Xu H , Zhang N , Yang D‐W , et al. Scan time reduction in intravoxel incoherent motion diffusion‐weighted imaging and diffusion kurtosis imaging of the abdominal organs: Using a simultaneous multislice technique with different acceleration factors. J Comput Assist Tomogr. 2021;45(4):507‐515. 34270482 10.1097/RCT.0000000000001189

[16] Marrale M , Collura G , Brai M , et al. Physics, techniques and review of neuroradiological applications of diffusion kurtosis imaging (DKI). Clin Neuroradiol. 2016;26(4):391‐403. 26589207 10.1007/s00062-015-0469-9

[17] He Y , Rong Y , Chen H , et al. Impact of different b‐value combinations on radiomics features of apparent diffusion coefficient in cervical cancer. Acta Radiol. 2020;61(4):568‐576. 31466457 10.1177/0284185119870157

[18] Bianchini L , Santinha J , Loução N , et al. A multicenter study on radiomic features from T2 ‐weighted images of a customized MR pelvic phantom setting the basis for robust radiomic models in clinics. Magn Reson Med. 2021;85(3):1713‐1726. 32970859 10.1002/mrm.28521

[19] Su C , Chen X , Liu C , et al. T2‐FLAIR, DWI and DKI radiomics satisfactorily predicts histological grade and Ki‐67 proliferation index in gliomas. Am J Transl Res. 2021;13(8):9182‐9194. 34540034 PMC8430185

[20] Tan Y , Mu W , Wang X‐C , Yang GQ , Gillies RJ , Zhang H . Whole‐tumor radiomics analysis of DKI and DTI may improve the prediction of genotypes for astrocytomas: a preliminary study. Eur J Radiol. 2020;124:108785. 32004731 10.1016/j.ejrad.2019.108785

[21] Sun K , Jiao Z , Zhu H , et al. Radiomics‐based machine learning analysis and characterization of breast lesions with multiparametric diffusion‐weighted MR. J Transl Med. 2021;19:1‐10. 34689804 10.1186/s12967-021-03117-5 PMC8543912

[22] Gitto S , Bologna M , Corino VDA , et al. Diffusion‐weighted MRI radiomics of spine bone tumors: feature stability and machine learning‐based classification performance. Radiol Med. 2022;127(5):518‐525. 35320464 10.1007/s11547-022-01468-7 PMC9098537

[23] Granzier RWY , Ibrahim A , Primakov S , et al. Test–retest data for the assessment of breast MRI radiomic feature repeatability. J Magn Reson Imaging. 2022;56(2):592‐604. 34936160 10.1002/jmri.28027 PMC9544420

[24] Traverso A , Kazmierski M , Welch ML , et al. Sensitivity of radiomic features to inter‐observer variability and image pre‐processing in apparent diffusion coefficient (ADC) maps of cervix cancer patients. Radiother Oncol. 2020;143:88‐94. 31477335 10.1016/j.radonc.2019.08.008

[25] Dreher C , Kuder TA , König F , et al. Radiomics in diffusion data: a test–retest, inter‐ and intra‐reader DWI phantom study. Clin Radiol. 2020;75(10):798.e13‐798.e22. 10.1016/j.crad.2020.06.024 32723501

[26] Gourtsoyianni S , Doumou G , Prezzi D , et al. Primary rectal cancer: repeatability of global and local‐regional MR imaging texture features. Radiology. 2017;284(2):552‐561. 28481194 10.1148/radiol.2017161375 PMC6150741

[27] Gitto S , Corino VDA , Annovazzi A , et al. 3D vs. 2D MRI radiomics in skeletal Ewing sarcoma: feature reproducibility and preliminary machine learning analysis on neoadjuvant chemotherapy response prediction. Front Oncol. 2022;12:1016123. 36531029 10.3389/fonc.2022.1016123 PMC9755864

[28] Zhao B , Tan Y , Tsai W‐Y , et al. Reproducibility of radiomics for deciphering tumor phenotype with imaging. Sci Rep. 2016;6:23428. 27009765 10.1038/srep23428 PMC4806325

[29] Schurink NW , van Kranen SR , Roberti S , et al. Sources of variation in multicenter rectal MRI data and their effect on radiomics feature reproducibility. Eur Radiol. 2022;32(3):1506‐1516. 34655313 10.1007/s00330-021-08251-8 PMC8831294

[30] Fasmer KE , Hodneland E , Dybvik JA , et al. Whole‐volume tumor MRI radiomics for prognostic modeling in endometrial cancer. J Magn Reson Imaging. 2021;53(3):928‐937. 33200420 10.1002/jmri.27444 PMC7894560

[31] Zhang Q , Yu X , Ouyang H , et al. Whole‐tumor texture model based on diffusion kurtosis imaging for assessing cervical cancer: a preliminary study. Eur Radiol. 2021;31(8):5576‐5585. 33464399 10.1007/s00330-020-07612-z

[32] Zhao X , Wang X , Zhang B , et al. Classifying early stages of cervical cancer with MRI‐based radiomics. Magn Reson Imaging. 2022;89:70‐76. 35337907 10.1016/j.mri.2022.03.002

[33] Wang M , Perucho JAU , Tse KY , Chu MMY , Ip P , Lee EYP . MRI texture features differentiate clinicopathological characteristics of cervical carcinoma. Eur Radiol. 2020;30(10):5384‐5391. 32382845 10.1007/s00330-020-06913-7

[34] Barth M , Breuer F , Koopmans PJ , Norris DG , Poser BA . Simultaneous multislice (SMS) imaging techniques. Magn Reson Med. 2016;75(1):63‐81. 26308571 10.1002/mrm.25897 PMC4915494

[35] Zahneisen B , Ernst T , Poser BA . SENSE and simultaneous multislice imaging. Magn Reson Med. 2015;74(5):1356‐1362. 25376715 10.1002/mrm.25519 PMC4420716

[36] McNabb CB , Lindner M , Shen S , Burgess LG , Murayama K , Johnstone T . Inter‐slice leakage and intra‐slice aliasing in simultaneous multi‐slice echo‐planar images. Brain Struct Funct. 2020;225(3):1153‐1158. 32140847 10.1007/s00429-020-02053-2 PMC7166208

[37] Lee KJ , Wild JM , Griffiths PD , Paley MN . Simultaneous multislice imaging with slice‐multiplexed RF pulses. Magn Reson Med. 2005;54(4):755‐760. 16155891 10.1002/mrm.20643

[38] Runge V , Richter JK , Heverhagen J . Simultaneous multi‐slice—a concise review covering major applications in clinical practice. Clinical Oncological Imaging. 2017;68(2):96‐101.

